# A service mapping exercise of four health and social care staff mental health and wellbeing services, Resilience Hubs, to describe health service provision and interventions

**DOI:** 10.1186/s12913-024-10835-1

**Published:** 2024-04-04

**Authors:** Kate Allsopp, Filippo Varese, Paul French, Ellie Young, Hannah White, Priscilla Chung, Jessica Davey, Alysha A. Hassan, Sally-Anne Wright, Alan Barrett, Gita Bhutani, Katherine McGuirk, Gemma Shields, Fay Huntley, Joanne Jordan, Aleix Rowlandson, May Sarsam, Hein Ten Cate, Holly Walker, Ruth Watson, Jenni Willbourn, Daniel Hind

**Affiliations:** 1 Complex Trauma & Resilience Research Unit, Greater Manchester Mental Health NHS Foundation Trust, Manchester Academic Health Science Centre, Research and Innovation, 3rd Floor Rawnsley Building, Hathersage Road, Manchester, UK; 2 School of Health Sciences, Faculty of Biology, Medicine and Health, Manchester Academic Health Science Centre, University of Manchester, Oxford Road, Manchester, UK; 3 Faculty of Health, Psychology and Social Care, Manchester Metropolitan University, Brooks Building, Bonsall Street, Manchester, UK; 4 Pennine Care NHS Foundation Trust, Trust Headquarters, 225 Old Street, Ashton-Under-Lyne, Greater Manchester UK; 5 Greater Manchester Resilience Hub, Pennine Care NHS Foundation Trust, Trust Headquarters, 225 Old Street, Ashton-Under-Lyne, Greater Manchester UK; 6 Lancashire and South Cumbria Resilience Hub, Lancashire and South Cumbria NHS Foundation Trust, Sceptre Point, Sceptre Way, Walton Summit, Preston, UK; 7 Humber and North Yorkshire Resilience Hub, Tees Esk and Wear Valleys NHS Foundation Trust, West Park Hospital, Edward Pease Way, Darlington, UK; 8 Cheshire and Merseyside Resilience Hub, Mersey Care NHS Foundation Trust, V7 Building, Kings Business Park, Prescot, UK; 9 School of Health Science, University of Salford, Mary Seacole Building, Frederick Road Campus, Broad St, Salford, UK; 10 Greater Manchester Health and Social Care Partnership, 4th Floor, 3 Piccadilly Place, Manchester, UK; 11 Doctorate of Clinical Psychology, School of Health in Social Science, Old Medical School, University of Edinburgh, Teviot Place, Room 2.2, Doorway 6, Edinburgh, UK; 12 Greater Manchester Mental Health NHS Foundation Trust, Trust Headquarters, Bury New Road, Prestwich, Manchester, M25 3BL UK; 13 School of Health and Related Research, The Innovation Centre, University of Sheffield, 217 Portobello, Sheffield, UK

**Keywords:** Service mapping, Mental health services, Healthcare staff, Implementation

## Abstract

**Background:**

NHS England funded 40 Mental Health and Wellbeing Hubs to support health and social care staff affected by the COVID-19 pandemic. We aimed to document variations in how national guidance was adapted to the local contexts of four Hubs in the North of England.

**Methods:**

We used a modified version of Price’s (2019) service mapping methodology. Service level data were used to inform the analysis. A mapping template was adapted from a range of tools, including the European Service Mapping Schedule, and reviewed by Hub leads. Key data included service model; staffing; and interventions. Data were collected between March 2021 – March 2022 by site research assistants. Findings were accuracy-checked by Hub leads, and a logic model developed to theorise how the Hubs may effect change.

**Results:**

Hub goals and service models closely reflected guidance; offering: proactive outreach; team-based support; clinical assessment; onward referral, and rapid access to mental health support (in-house and external). Implementation reflected a service context of a client group with high mental health need, and high waiting times at external mental health services. Hubs were predominantly staffed by experienced clinicians, to manage these mental health presentations and organisational working. Formulation-based psychological assessment and the provision of direct therapy were not core functions of the NHS England model, however all Hubs incorporated these adaptations into their service models in response to local contexts, such as extensive waiting lists within external services, and/or client presentations falling between gaps in existing service provision. Finally, a standalone clinical records system was seen as important to reassure Hub users of confidentiality. Other more nuanced variation depended on localised contexts.

**Conclusion:**

This study provides a map for setting up services, emphasising early understandings of how new services will integrate within existing systems. Local and regional contexts led to variation in service configuration. Whilst additional Hub functions are supported by available literature, further research is needed to determine whether these functions should comprise essential components of staff wellbeing services moving forward. Future research should also determine the comparative effectiveness of service components, and the limits of permissible variation.

**Study registration:**

researchregistry6303.

## Background

The COVID-19 pandemic impacted the mental health of health and social care workers, with staff working in frontline roles and critical care particularly affected, [1, 2] especially﻿﻿﻿ or those redeployed into new roles [3]. Staff from Black, Asian, and other diverse ethnic communities [4], staff with existing physical or mental health conditions, and those with limited social support were also at high risk of mental health difficulties [2, 5–8]. Internationally, 49% reported insomnia, 47% anxiety, and 37% post-traumatic stress [9]. Levels of distress remained high two years after the start of the pandemic [5]. The impact from pandemics lasts for years, [10, 11] highlighting the need for sustainable mental health support services for health and social care staff.

'Screen and refer’ models aim to quickly identify need, and provide access to evidence-based interventions, to mitigate symptoms and the risk of longer-term difficulties following disasters [12]. One such model, the Greater Manchester Resilience Hub, found those who completed screening immediately after the 2017 Arena bombing had better outcomes that those who screened nine months after the incident [13, 14]. In October 2020, NHS England and Improvement (NHSE&I) funded 40 resilience hubs based on this service model to support staff during the pandemic [15].

NHSE&I guidance for Hubs in 2020 allowed variability in implementation, [16] but defined key service components as: 1) proactive outreach; 2) building capacity via team-based training; 3) rapid clinical assessment; 4) onward referral to coordinate rapid access to mental health support. What is not clear is how funded services operationalised these guidelines locally and how they varied in the support provided [17]. Evaluating such services means establishing not only clinical effectiveness, but also context-dependent features—which components are critical, which are adaptable, and how the intervention produces change [18]. Understanding how services may have varied in their implementation is a vital part of identifying key aspects for evaluation in future pandemics or disasters.

Different service mapping methods are available to describe and classify health services, [19] from macro-quantitative approaches for international comparisons, [20] to more granular assessments [21]. We undertook a small-scale mapping exercise, to describe how four resilience hubs in the North of England implemented the NHSE&I model.

## Methods

We adapted Price’s steps to mapping services: [22].

1. *Define services*: Four mental health and wellbeing staff hubs funded by NHSE&I to support health and social care staff affected by the pandemic. These services were the four Hubs involved in the wider mixed-methods evaluation of the Resilience Hub model, and were selected as they were four of the earliest Hubs to become operational during the pandemic.
2. *Determine sources of information:* Service level data was provided by authors AB; JW; RW; KM; GB; HTC; MS; FH; HW; JJ who were Hub service managers and clinical leads. Additional sources included business cases and hub websites.
3. *Survey design:* A service mapping template (Table 1), using free text response boxes, was synthesised from three published instruments: (a) Sections A and D of the European Service Mapping Schedule [19, 23]; (b) the Template for Intervention Description and Replication (TIDieR) checklist [24]; (c) a checklist for describing health service interventions [25] for prompts about organisations, workforce and staffing.
4. *Data collection:* Research Assistants (RAs) compiled template data provided by paper authors (service managers and clinical leads), during financial year 2021–22. Inaccuracies in completed templates were checked for and resolved through emails and meetings with relevant paper authors.
5. *Data analysis:* Data were integrated to compare and contrast features across sites. The summary document was reviewed individually by all four site clinical leads to ensure the accuracy of integrated service descriptions, and as a team at two group meetings, again, with all four site leads. To protect anonymity, the participating sites are referred to as Sites A-D.

**Table 1:** Service mapping components and definitions

Service mapping component	Definition
*Contextual Information*	
Population of in-scope staff by region	Estimated numbers of in-scope health and social care staff across site regions
*Service inventory*	
Goals and model summary	Goals of Hubs and overview of service model
Target population	Occupational groups eligible to receive support from the Hubs
Workforce and staffing	Staffing groups, skill mix, and full time equivalents
Funding	Funding source
*Interventions and services provided by Hubs*	
Outreach and promotion	Methods of outreach and promotion to encourage support uptake
Universally available support (website resources)	Self-help resources available on Hub websites
Self-referral and mental health screening	Overview of referral and screening tools
Clinical assessment and formulation	Method of psychological assessment
Support for Individuals	Types of support available for individuals, including conditions under which onward referrals were made out of the service, and extent to which this was used
Support for teams	Support available for staff teams
Other support	Additional support available, not already encompassed above

### Communication of findings

A logic model, to show how the Hubs may effect change, was developed. The staff wellbeing hub model was based on that of the Greater Manchester Resilience Hub, therefore the starting point for the logic model from this paper was based on prior work at this Hubs [13]. The model developed rapidly during the course of this study, and amendments to the logic model were led by the team’s health economists (GS and AR) in alignment with the shape of economic work developed within the health economic component of the wider mixed-methods study. The logic model was also considerably developed through feedback from all four Hubs involved in the study. It is used here to illustrate the overall model of the services described.

## Results

### Contextual information

#### Population of in-scope staff by region

Table 2 outlines the approximate number of health and social care staff working within each region eligible for Hub support.

**Table 2:** Number of health and social care staff in each region

Site	Health and social care staff within site regions *(excluding staff from private organisations)*
Site A	126,000
Site B	165,000
Site C	129,000
Site D	180,000

### Service inventory

#### Goals and model summary

Hubs aimed to offer timely psychological support to health and social care staff who had been affected by the pandemic, at individual, team, and organisational levels. The function of the Hubs continued to evolve, broadening beyond the pandemic, e.g., providing support following local incidents. Each Hub used online self-referral, gathering mental health, demographic and occupational information, which informed subsequent clinical assessment. Most support was provided virtually, with expansion to face-to-face support as COVID restrictions eased, Monday–Friday, with some evening appointments available.

#### Target population

All Hubs supported staff from the NHS and social care, including third sector and local authority (Table 3). Some extended care to key workers outside of the NHSE&I national scope, including from education and non-ambulance emergency services. NHSE&I-defined eligibility also changed over time [26]. Site A opened to all staff in December 2020. Other Hubs had a phased extension of care to successive occupational groups to ensure demand was met. Site B opened to critical care staff in February 2021, other NHS staff in March, social care staff in July and non-NHS emergency services in August. At Site C, a pilot service enhanced occupational health from November 2020 and was scaled up in February 2021. Site D opened screened and referred targeted NHS groups from May 2020, and all health and social care staff and their families from November.

**Table 3:** Groups in scope for Hub support at each site

	**Site A**	**Site B**	**Site C**	**Site D**
**Over 18** (health & care staff)	✓	✓	✓	✓
**16–17 years** (health & care staff)	✓	Not in scope	✓	✓
**Family members** ^a^	✓	Not in scope	✓	✓
**Ambulance** **Service**	✓	✓	✓	✓
**Police/Fire**	✓	✓	✓	✓ (if involved in specific COVID-related health & care duties)
**Education**	✓	Not in scope	Not in scope	✓ (if responsible for wellbeing)
**3rd Sector** ^b^	✓	✓	✓	✓
**VCSE**	✓	✓ (if local authority commissioned)	✓	✓

^a^Definition of family members differed across sites. Within Site C and Site D, family members referred to both immediate and chosen family living in any location. Within Site A, family members referred to immediate family, including in-laws, who must live in the Hub’s region

^b^Social care; local authority-funded; private health and care

### Workforce and staffing

A range of staff were employed by Hubs. Table 4 outlines the Hub staff types and their whole-time equivalents (WTEs).

**Table 4:** Hub staffing numbers in post at the time of data collection (March 2021-March 2022), expressed as Whole Time Equivalents (WTEs^a^)

Role	AfC Banding	Site A	Site B	Site C	Site D
Clinical leadership	8b-9	1.2	2.6	1	2.15
Service / business manager and strategic engagement roles	6-8a	0.4	2.7	2	1.5
Psychological therapists and psychologists	6-8b	7.7	12.5	8.5	5.4
Pharmacist/ Non-Medical Prescriber	8a	0.6	-	-	-
Staff offering psychoeducation, low intensity interventions, and pastoral care (e.g. assistant psychologists)	4–5	1	7.8	5.6	2.1
Research staff	5–6	1.4	0	1	0
Administrators	3–5	1.6	1	1.8	1
Total WTEs		13.9	26.6	19.9	12.15

Detailed breakdown available on request

^a^1.0 WTE is the equivalent of a 37.5 h full time working week

Hubs all used a ‘top-loaded’ model with a higher number of senior clinicians compared with non-qualified staff such as Assistant Psychologists (APs). Table 5 shows the proportion of staffing at NHS Band 6 (clinically qualified or trainee clinical psychologists) and above. Staff were employed via secondment or fixed-term contracts.

**Table 5:** Proportion of qualified clinicians at each Hub

Service	Percentage of Band 6 staff or higher*
**Site A**	78.51%
**Site B**	67.04%
**Site C**	73.96%
**Site D**	74.17%

^*^The proportion of qualified staff at the time of data collection, according to NHS Agenda for Change (AfC) banding. Band 6 generally corresponds to qualified clinicians

More senior staff were employed in anticipation of the need to support teams and organisational working, individuals with moderate to severe mental health difficulties, and to ensure accreditation/experience in NICE-approved trauma-focused interventions [27]. Each Hub’s triage assessment and treatment planning was led by psychological formulation; ensuring a collaborative approach to explain difficulties and make sense of them whilst acknowledging the individual’s strengths and resources, [28] necessitating experienced clinicians skilled in this type of assessment. Workforce shortages mean that the reported staffing numbers, grading and roles (Table 4) fell considerably short of those planned in Hub business cases.

#### Funding

Each Hub was funded by NHSE&I, with some variation in local funding arrangements. The Hubs were each hosted by one NHS Trust but represented collaborations between several trusts within their respective localities.

### Interventions and services offered by the Hubs

#### Outreach and promotion

Promotional outreach was recommended by the NHSE&I guidance and seen by Hub leads as an important part of the Hubs’ work, to overcome stigma and increase uptake. Methods included: meetings with health and care leadership; presentations to teams; distribution of publicity materials; and local media campaigns. Notable variations included the use by Site D of a locality system, assigning Hub clinicians to specific workforces/areas and the employment by Site B of a full-time Strategic Engagement Lead. Methods to reach staffing and demographic groups with lower uptake included: a race equality campaign (Site B); visiting and providing materials to care homes (Sites B, C and D); producing information for care homes (Sites A and D); gathering email addresses to promote the offer to care home, ambulance and hospice staff (Site C; Site D); bespoke social media graphics (Site B; Site D); and, promoting workshops / facilitated peer support sessions for care home staff (Site B, C and D) and men (Site C; Site D). Meetings and webinars were conducted with targeted groups, including: equality leads and race equality networks (Sites C and D); the local council of Mosques (Site A); and, emergency services (Sites A and D).

#### Universally available support

Each Hub had a website, providing information about services, eligibility, psychoeducation materials, and downloadable self-help resources and short webinars. Websites provided details of mental health services/charities available to staff, crisis helplines for emergencies, and enabled keyworkers to self-refer to Hubs.

#### Self-referral and mental health screening

Standalone online clinical records systems, enabling self-referral, and mental health/demographic survey instruments, [29–34] were not identified in NHSE&I guidance, but were used with the rationale of: reassuring client groups of confidentiality; allowing triage, monitoring of severity, population reach, and research. Online mental health questionnaires were used: as self-assessment tool with immediate feedback and the option to self-refer to the Hub (Site A); after acceptance of self-referral, prior to clinical assessment (Site B), and, as part of self-referral into the service, with immediate feedback via email (Sites C and D). See Table 6 for details of questionnaire measures used at each site.

**Table 6:** Screening measures utilised at each Hub

	**Site A**	**Site B**	**Site C**	**Site D**
**Demographic and occupational questions**	✓	✓	✓	✓
**PHQ-9**	✓	✓	✓	✓
**GAD-7**	✓	✓	✓	✓
**WSAS**	✓	✓	✓	✓
**AUDIT**	✓	no	✓	✓
**ITQ**	no	no	✓	✓
**PCL-5**	✓	✓	no	no
**Smoking/Drug use**	✓	✓	✓ (Since September 2021)	✓ (Since May 2021)
**Questions around the impact of COVID-19**	✓	✓	✓	✓

#### Clinical assessment and formulation

Rapid access to clinical assessment, one of NHSE&I’s core Hub functions, was available at all sites and informed by mental health screening data. Sites C and D offered a two-stage assessment process of a shorter assessment followed by an in-depth clinical assessment if clinically indicated, whereas Sites A and B offered in-depth clinical assessment as standard. Shorter assessments at Sites C and D were offered by all staff grades, lasted 30–60 min, informed by questionnaire data, and aimed to agree evidence-based support.

In-depth assessments were delivered by a qualified cognitive behavioural therapist or clinical psychologist. Assessments followed a pre-determined clinical framework led by psychological formulation, and were conducted by senior clinicians. If risk was a concern (e.g., self-report of suicidal ideation or self-harm), duty clinicians at the Hubs further assessed the nature of risk and level of distress, and facilitated support and/or access/referral to appropriate services. Formulation-led psychological assessment was not specified by NHS England as a core Hub function, however all four Hub clinical teams made this approach available in order to best understand the needs of clients who had more severe mental health needs, or complexity in terms of more enduring difficulties or co-occurring difficulties across multiple domains.

#### Support for individuals

##### Onward referrals

An NHSE&I core function of Hubs was to facilitate onward referral and navigation to further support, including therapy where clinically indicated.

At Sites B, C, and D the majority of clients were referred to other mental health services to maximise usage of existing services, following assessment, evidence-based psychologically informed advice, self-help support and psychoeducation as needed. Where required, outreach and clinical advocacy was used to support clients to access external services. Onward referral was facilitated between Site D and the wider system by using facilitated assessments to prevent clients from having to retell their story in multiple services.

NHSE&I did not prescribe direct therapy as a core function, but all Hubs provided this level of support to some extent. Where Hubs saw clients for direct therapy in-house, the rationale was similar across sites, to meet the needs of: clients with a high level of mental health need or multiple co-occurring difficulties; clinical risk factors otherwise unmet; unavailability of timely therapy in the wider mental health/staff support system. At Site A, unavailability of therapy in mainstream mental health services, due to limited service availability and lengthy waiting times, drove an adaptation of the model at this Hub to provide the majority of clients with in-house therapy. Consequently, only a small number of onward referrals were made to other services.

##### Self-Help & Psychoeducation

Self-help, guided self-help, and psychoeducation were offered at all sites, including information materials, explanations of difficulties (e.g. why difficulties such as anxiety or low mood may develop and what keeps them going), and signposting to other services, with resources available on Hub websites. There were minimal variations across Hubs, for example some sites were more flexible around session numbers (Site D), and others offered more formalised low intensity interventions if clinically indicated (Sites A, B, and C). Site B also provided a pastoral care pathway involving engagement with community, charity and third sector organisations to offer an alternative model of care for interest-based support, such as martial arts or music groups.

##### Low intensity interventions

Sites A, B and C provided low intensity (Step 2) interventions for mild to moderate difficulties, which was more formally structured than the self-help and psychoeducation offered at Site D. Low intensity interventions were usually semi-structured or manualised, based on low intensity CBT principles, and covered a range of topics such as sleep, anxiety, or panic. Across sites, an average of six sessions were delivered by a range of clinicians but typically APs under clinical supervision, or associate psychological practitioners. Following intervention, clients could be ‘stepped up’ to high intensity therapy if clinically necessary, referred on, or discharged.

##### High intensity interventions

As described above, direct therapy was offered by all Hubs as a central component of support. The rationale for providing direct therapies was similar across Hubs, including: significant waiting times at local services; particular types of complexity (e.g., concerns around confidentiality; previous negative experiences in services) and circumstances in which clients’ presentations fell between gaps in services (e.g., difficulties that were too complex for IAPT services, but not sufficiently complex or enduring for Community Mental Health Team (CMHT) support).

High intensity therapy was delivered by CBT Therapists, consultant practitioners and CPs trained in EMDR and trauma-focused CBT, and modalities included for example, CBT; Cognitive Analytic Therapy (CAT); Compassion Focused Therapy (CFT); Acceptance and Commitment Therapy (ACT); and trauma-focused interventions such as trauma-focused CBT and EMDR. This usually consisted of approximately 12 sessions at each Hub.

Minor variations across Hubs included further dividing high intensity therapies into ‘Step 3’ (8–12 sessions) and ‘Step 4’ interventions (20 + sessions) dependent on the severity or clinical complexity of presenting difficulties (Sites B and D). Sites B and C also offered group therapies delivered by qualified clinicians, providing support for bereavement and Long COVID.

##### Pharmacological intervention

Site A was the only Hub to offer pharmacological advice, an offer outside of the core NHSEI Hub functions. Pharmacological intervention was delivered by pharmacists, including psychoeducation, new mental health prescriptions, and medication review. Site C offered medication support from a non-prescribing associate nurse consultant, including psychoeducation, advice, and GP liaison.

#### Support for teams

Team-based support represented the final core Hub function outlined by NHSE&I, and was seen as important by Hubs for proactive system-preparedness, raising awareness, and increasing access to individual support. A wide range of team-based interventions was offered across Hubs by clinical leads, clinical psychologists or other psychological practitioners, developed to support to the needs of managers, team leaders, and help support the psychological safety of the health and social care system.

Whilst there were variations across sites, all sites provided consultation with managers and team leaders to help identify difficulties faced by teams, such as systemic challenges, and provide bespoke solutions. Team-based work incorporated trauma-informed approaches, signposting to supportive resources, reflective sessions, self-care workshops, training for teams, and organisational strategy support, as well as direct support for managers as needed.

Workshops were provided virtually and face-to-face where appropriate across the Hubs. These were delivered by qualified clinicians with experience in supporting teams and organisations, and focused on different emotional wellbeing, self-care, psychological first aid, and validation of the team’s difficulties. Furthermore, facilitated peer support was also offered by some Hubs to provide a safe reflection space for teams to come together to discuss relevant topics/difficulties.

Team-based interventions were bespoke to teams’ needs as determined through initial consultation and formulation, and could comprise different interventions; an example is given in Table 7.

**Table 7:** Example of team-based support offered by one Hub

*A team manager contacted the Hub for support, and details of the Hub were sent to all services within remit in the Hub’s region * ***(promotion of the offer).*** * An initial discussion with the team manager identified current difficulties * ***(consultation).*** * The manager provided the Hub with staff email addresses, who were sent information on screening/self-referral and the Hub’s offer * ***(outreach)***	
*Face-to-face workshops were offered to this and other local teams offer solutions and build psychological safety * ***(workshops).*** * The Hub joined the team’s ‘diversity and inclusion group’ to ascertain potential barriers to service access. A group of ward managers requested a facilitated peer support session to validate experiences and provide an opportunity for reflection * ***(facilitated peer support)***	
*Following this, the team experienced a death within their service, which resulted in the Hub * ***re-promoting the offer*** * and explained to staff how to re-engage with the Hub without having to “re-register” or complete questionnaires. Staff were signposted to a bereavement service to provide more specialised support * ***(onward referral)*** *. Further * ***consultation, promotion of support*** * and a face-to-face * ***workshop*** * were provided. The team could also contact the Hub for support for additional difficulties*	

#### Other support

Three of the Hubs utilised additional available funding to provide and coordinate support for critical care staff, determined according to the number of ICU beds taken up with COVID-19 patients. There were minor variations, but Hubs coordinated to provide broadly similar support across the services, including: promotion of support; wellbeing support for individuals, teams, and particular staffing groups (such as more senior staff and professional nurse advocates); training; and consultation.

**Fig. 1:**
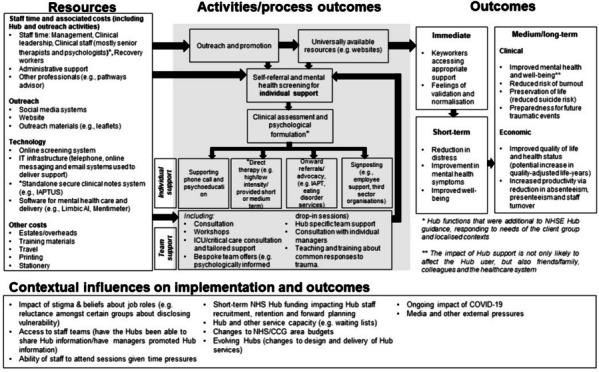
Logic model detailing the Resilience Hub model and how its outcomes are produced

Figure 1 presents a logic model, further developed as part of this mapping exercise, to incorporate an overview of components of the Hub service model described above, and provide an understanding of how the Hub model produces change.

## Discussion

This paper details the service models of four resilience hubs set up to support health and social care staff affected by the pandemic. Hub service models offered NHSE&I-described core functions including: proactive outreach; team-based support; clinical assessment; onward referral, and coordinated rapid access to mental health support.

Implementation varied with localised contexts, and four additional functions were added at all four sites: 1) standalone clinical record systems to reassure keyworkers of confidentiality; 2) psychological formulation-led assessment; 3) provision of direct therapy; 4) a clinical team of predominantly senior therapists and psychologists. Within the model of these four Hubs, senior clinicians were considered important to: support teams; support people with complex needs (e.g. multiple, severe, and/or enduring mental health difficulties); and, psychological formulation-based assessment, to sufficiently assess need and conduct treatment planning. Sites varied in the proportions of outward referrals to local mental health services; provision of direct therapy became a core component for all Hubs, to address: significant waiting times; clinical complexity (see above); and, circumstances in which clients’ presentations fell into gaps between services.

These four additional Hub functions, representing adaptations from the NHSE&I guidance, are supported by findings from the qualitative component of the wider mixed methods study, [35] including the value placed by keyworkers on a confidential service, independent from occupational health services, and the view of wider stakeholders (e.g. HR leads, occupational health leads) that the Hubs were best placed to support staff with more severe mental health needs, as they were able to offer high intensity interventions including trauma therapies. The adaptations are also supported by a report published by the British Psychological Society (BPS) outlining best practice principles for staff wellbeing support, derived from literature review from other hubs, Freedom of Information requests to NHS England and local commissioners, and an expert roundtable with Hub leads across England [36]. Supporting principles from the BPS report include: independent and confidential services;(see also [37]) psychologically-informed services, including ‘reversal of the stepped care model’, or direct access to experienced therapists/ psychologists from the first appointment, compared with ‘stepped care’ models within the NHS whereby support is initially delivered by more junior staff and ‘stepped up’ to senior therapists if indicated. Finally, the updated NHSE&I Hub guidance published in 2022 supported Hubs to provide interventions directly to fill service gaps and thereby meet local need, which in part supports the direct provision of psychological interventions at the Hubs described in this paper [26].

However, these adaptations nevertheless require further evaluation to understand whether these components should be an essential part of the core offer of staff wellbeing services moving forward. Whether there are any inadvertent implications of these additional functions should also be explored, for example, the systemwide impact on staffing across mental health services if more senior therapist and psychologists are seconded into staff wellbeing hubs.

The Hubs’ key components—proactive outreach, psychoeducation, and a stepped-care approach to support—were all consistent with recommendations from the disaster literature [38]. Their multi-layered support offer appears substantial in comparison with Employee Assistance Programmes typically offered through organisations such NHS Trusts, which are frequently deemed insufficient by staff, [39] and other low intensity interventions available during the pandemic (e.g. [40]). The COVID-19 literature supports the Hubs’ use of standalone clinical systems to reassure staff of confidentiality, the absence of which are a barrier to help-seeking [37, 41]. The literature highlights the importance of proactive approaches and positive supportive work environments, [39, 41] consistent with the Hubs’ team-based support, while suggesting that improvements are needed to workplace cultures that do not rely on external services. Another barrier that could be more proactively addressed is the availability of flexible interventions that can be accommodated within workplaces, such as peer support, or protected time for staff to attend [41]. The impact of pandemics on healthcare staff, including the recurring threat of infectious disease, is pervasive, and highlights the importance of a prepared workforce, with Hub-like support services and systemic work to improve workplace culture and flexible access [42].

We used a modified version of Price’s method of service mapping; this paper represents the final two steps, communication of findings and hosting of the logic model. The four study sites represent only 10% of the 40 wellbeing hubs funded by NHSE&I, and were involved in the wider study due to the similarities in their service model, therefore the findings may not reflect the full range of variations to the Hub model nationally. Adaptation of research methods, the findings and interpretation, were co-produced with senior management from the involved sites, assuring the internal validity of findings. Comparisons of the findings with other sources of data and a wider range of informants, for example from the qualitative component of the wider mixed methods study, and data from other staff wellbeing hubs, [37] were employed to reduce the potential for bias and increase the trustworthiness of the paper.

This mapping exercise offers further groundwork for the evaluation of Hub effectiveness during future pandemics or disasters, however further research is needed to support our logic model and integrate into it effectiveness, outcomes, and process data. Such data would help to determine which Hub functions were essential and/or warranted, for example, the proportion of clients requiring an in-depth psychological formulation-led assessment, and in what time-frame. For example, at the time of writing, no definition was available for NHSE’s guidance to provide ‘rapid assessment’, but process data could further explore waiting times for assessment and intervention compared with other services.

Given the Hubs were services set up to support populations following major incidents or crises, a randomised trial would face considerable pragmatic and ethical challenges. Instead, a large-scale naturalistic evaluation using a quasi-experimental design would enable a comparison of the outcome data for health and social care staff in regions where Hub support is available, compared with regions where Hub support is not available, to determine the clinical and cost effectiveness of the model. By applying the service mapping tool to additional sites involved in a large-scale evaluation, this design could also be used to determine the comparative effectiveness of service components, the extent to which they represent critical functions, and to determine the limits of permissible variation.

In conclusion, Hub service models reflected NHSE&I guidance, but with some significant local adaptations. Components that were additional to the NHSE&I guidance were utilised at all four Hubs in response to local contexts. Whilst these additional Hub functions are supported by available literature, further research is needed to determine whether these functions should comprise essential components of staff wellbeing services moving forward. Examination of how services will integrate with existing mental health and staff support systems is an important consideration in the development of service models. As the study examined a small proportion of NHSE&I-funded services, these findings may not represent the national picture of staff wellbeing hubs. Further research is needed to evaluate the effectiveness of the Hub model against compared with other staff support, to identify critical components, and understand the limits within which the model can be adapted.

## Data Availability

All data requests should be submitted to Kate.Allsopp@gmmh.nhs.uk for consideration. Access to anonymised data may be granted following review in consultation with broader study team and Sponsor.
